# A Review of the Literature on Surgical Management in the Acute Phase of Spinal Cord Injury: Expected Outcomes and the Influence of Surgical Timing

**DOI:** 10.7759/cureus.84066

**Published:** 2025-05-13

**Authors:** Anastasios Kalampokis, Ioannis S Benetos, Elias Vasiliadis, Spyros G Pneumaticos

**Affiliations:** 1 Orthopaedics, KAT Hospital, Athens, GRC; 2 Orthopaedic Surgery, National and Kapodistrian University of Athens, KAT Hospital, Athens, GRC

**Keywords:** influence of timing, neurologic outcomes and complications, spinal cord injury, surgical management vs. time, surgical timing

## Abstract

Certain mechanisms of injury to the spine can produce neurological impairment. Such phenomena are called spinal cord injuries (SCI). The management of SCI in the acute phase can prove challenging, especially in polytrauma patients, where an incomplete neurological deficit necessitates urgent surgical decompression. In order to investigate the expected outcomes and the influence of surgical timing in the surgical management in the acute phase of SCI, a systematic review of the literature was performed by examining online databases such as PubMed-NCBI, Web of Science, Cochrane Library, Scopus, and Embase to identify relevant scientific articles. Keywords (medical subject headings (MeSH) terms) used in the search included "spinal cord injury," "surgical decompression," "surgical timing," and "neurological outcomes”. Initially, 346 studies were identified, and after the application of the exclusion criteria, 43 studies were included in this review. The current body of literature supports early decompressive surgery, ideally within 24 hours of injury, as a guideline in the management of acute SCI. This timing is essential to raise the chances of neurological improvement in the presence of an incomplete SCI.

## Introduction and background

Improving patient outcomes and quality of life requires effective management of spinal cord injuries (SCI). Decompression surgery, in particular, is essential for reducing the mechanisms of secondary compressive phenomena that arise after the initial trauma [1]. The timing of surgery is a significant factor influencing recovery trajectories, with early decompression often associated with enhanced neurological outcomes [2]. Evidence increasingly suggests that interventions conducted within 24 hours post-injury can lead to significantly better prognoses. Studies, such as those by Fehlings et al. [3], emphasize the correlation between prompt surgical intervention and improvements in neurologic function, underscoring that timely decompression may reduce complications at the six-month follow-up.

Research has shown that each minute counts following an acute SCI in case of an incomplete injury. Gupta et al. found that decompression performed within 24 hours post-injury resulted in neurologic improvements for a greater percentage of patients than those treated later [4]. The association of early surgical treatment with a higher rate of recovery of anterior motor function has been supported by additional studies, although the nuances in their findings suggest that individual circumstances need to be taken into account [5]. On the other hand, the potential benefits of delayed interventions continue to be debated. Some evidence suggests that while immediate decompression tends to be optimal, beneficial outcomes may still arise from surgeries performed beyond the 24-hour window, for example, in the presence of neurogenic shock [6].

The evolving narrative surrounding surgical timing in acute SCI management necessitates ongoing examination. A progressive, deteriorating neurological image is a more urgent situation in comparison with a stable one. The current literature review highlights the need for a nuanced approach that considers individual patient scenarios, injury severity, and surgical risk factors [7,8]. More recent contributions further complicate the discourse, indicating varied outcomes associated with delayed surgeries and potential neuroprotective strategies [9]. This review aimed to synthesize the current understanding of the surgical management of SCI, focusing on identifying expected outcomes in comparison with the surgical timing while engaging with emerging perspectives in the field.

## Review

Materials and methods

**Figure 1:**
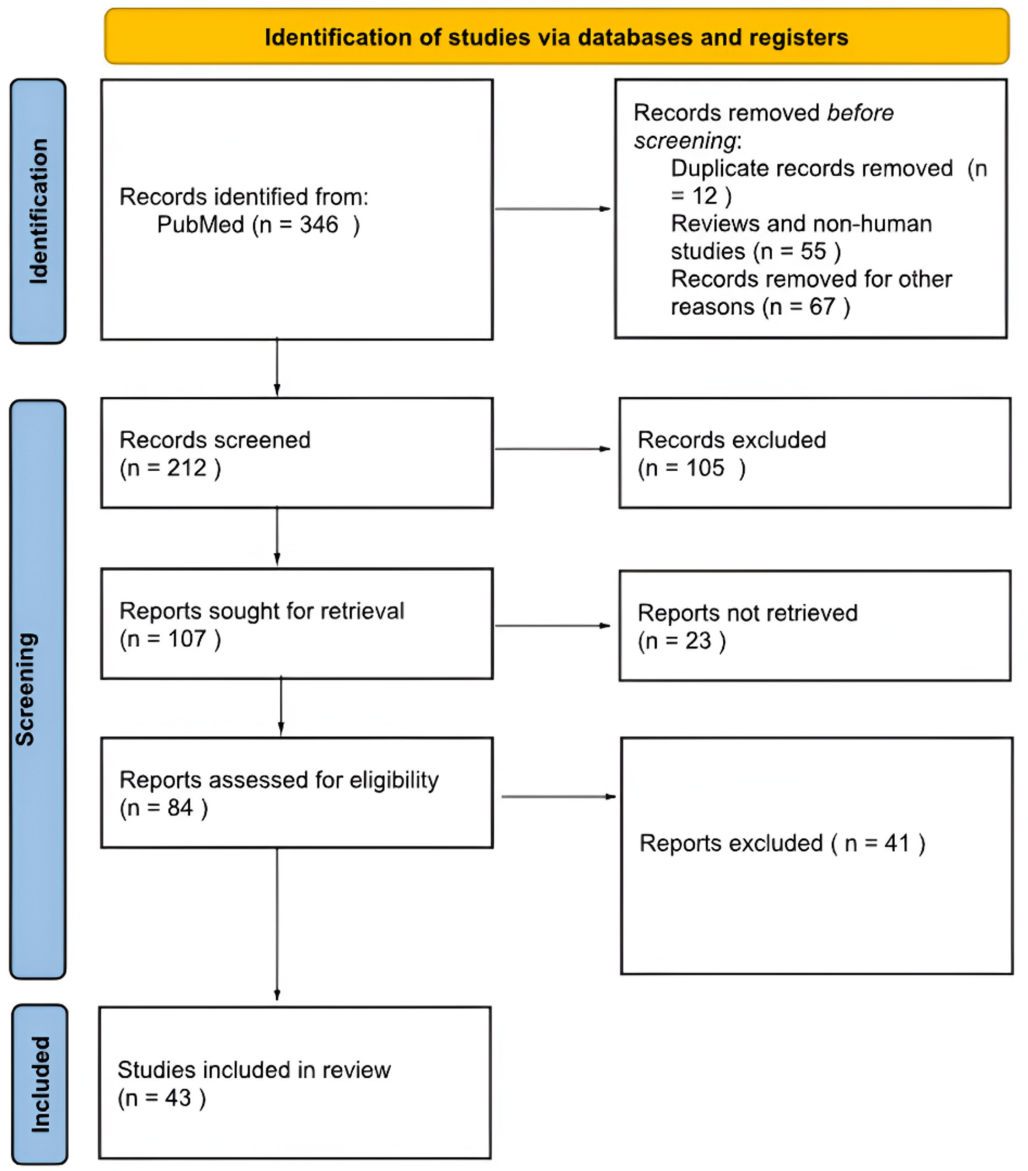
Flowchart on study selection

To conduct this systematic review, electronic databases such as PubMed, Embase, the Cochrane Library, and Web of Science were searched for articles using medical subject headings (MeSH) terms such as "spinal cord injury," "surgical decompression," "surgical timing," and "neurological outcomes." The review protocol was developed a priori per methodological standards outlined in previous literature to ensure transparency and reproducibility. Inclusion criteria were defined to encompass randomized controlled trials, observational studies, and systematic reviews evaluating surgical management in the acute phase of SCI with reported outcomes on functional and neurological recovery. Studies focusing exclusively on non-surgical or conservative treatment modalities or those failing to stratify outcomes based on surgical timing were excluded from further analysis. Initially, 346 studies were identified through the database, but after careful evaluation and exclusion, 43 studies were included in this review (Figure 1). This review aims to comprehensively evaluate the surgical management of SCI in the acute phase, taking into consideration factors such as surgical timing and neurological outcomes.

Results

Correlation Between Fracture Type and Neurologic Deficit

A variety of studies have evaluated the relationship between the type of spine fracture and the emerging neurological damage, while different fracture classifications show different patterns in neurological outcomes [10]. For example, neurological deficits have been consistently linked to burst fractures, which are characterized by multifragmentation of the vertebral body and retropulsion of bony fragments into the spinal canal. Studies suggest that 50% to 60% of patients with thoracolumbar burst fractures have neurological impairment [11]. The relationship between sagittal alignment, the rotation angle of bony fragments, and the degree of nerve damage has not been well documented, and no multivariate statistical analysis has taken into account all potential markers of neurologic damage or discovered methods for evaluating neurologic deficit using radiographic parameters. Four radiographic parameters, namely vertebral body compression, spinal canal stenosis (SCS), sagittal alignment, and reverse fragments, were selected for this investigation to evaluate neurologic deficit following thoracolumbar burst fracture [11].

Additionally, compression or wedge fractures tend to present with a lower risk of neural compromise, a finding supported by analyses correlating fracture morphology with neurological status [12]. Fontijne et al. discovered a significant correlation between neurological deficit and the percentage of SCS, but the severity could not be predicted; the degree of cervical and thoracic canal compromise attributed to retropulsed vertebral burst fractures/fragments positively correlated with the severity of the resulting neurological deficits. However, there was no correlation between the severity of cauda equina symptoms and lumbar burst fractures [13]. Moreover, studies employing CT scans have reinforced that the degree of spinal canal compromise in burst fractures is a significant predictor of the severity of neurological impairment [13].

Further research suggests that specific fracture subtypes within wider classifications carry different risks of neurological injury. For example, data from studies conducted in trauma centers indicate that while type A fractures (as classified by the AO Spine system) are the most common, they are frequently associated with neurological deficits, underscoring the need for careful evaluation during clinical assessment [14]. The neurologic deficit was more common in type A fractures at 44.9%. An observable neurologic deficit was seen in 40.6% of patients with type B fractures and 14.5% of individuals with type C fractures. There was a statistically significant correlation between the type of fracture and neurologic impairment [14]. In contrast, Chance fractures and flexion-distraction injuries, which typically result from high-energy trauma, have shown intermediate rates of neurological deficit, likely due to varying degrees of displacement and injury to the posterior elements. These correlations between fracture mechanism and neurological outcome provide a strong basis for further research and refinement of management algorithms in spinal trauma [15], as demonstrated in Table 1.

**Table 1:** Correlation between the type of injury and neurological impairment

Type of fracture	Incidence	Characteristic	Risk of neurologic deficit	Study
Burst fractures	55%	High canal compromise	Significant risk of neural injury	Tang et al. [11], Montes-Aguilar et al. [13]
Wedge compression	15%	Minimal retropulsion	Lower risk of neural involvement	Park et al. [12]
Chance fractures	30%	Associated with flexion-distraction forces	Moderate risk	Tang et al. [11]
Flexion-distraction Injuries	65%	Severe damage due to ligamentous disruption	High risk	Tang et al. [11]
C Translational Injuries	54.5%	Marked displacement	High risk	Khan et al. [15]

Timing of Surgical Intervention: Definitions and Classifications

In the spectrum of acute SCI, the definition of “early” surgical intervention has been variably characterized in the literature (Table 2), with thresholds ranging from ≤8 hours to ≤72 hours post-trauma. Ahuja et al. emphasize the importance of detailed care in acute management, noting that early decompression is associated with better neurological recovery [16]. Similarly, Balain (2017) discusses the role of surgery and emphasizes that physiologic stability and the injury’s characteristics often affect the classification of surgical timing. According to him, there is little evidence to justify early surgery within 24 hours, and there is no difference in the length of hospital or intensive care unit stays between groups that have early versus delayed surgery. In terms of neurological recovery, there was strong evidence that surgery performed within two weeks is preferable to surgery performed after two weeks. Additionally, while early surgical decompression is appropriate and safe for individuals with American Spinal Injury Association (ASIA) C, people with ASIA D impairment can be treated first with monitoring, followed by surgery scheduled for a later time based on recovery [17]. Thomas et al. further underscore that ultra-early interventions (defined as interventions performed within eight hours) are increasingly being examined as potential standards for improving clinical outcomes [18], while Wilson and Fehlings note that many studies commonly adopt a <24-hour threshold as a practical delineation for early surgery [19].

**Table 2:** Definitions and classifications of surgical timing in acute SCI SCI: Spinal cord injuries

Study	Year	Timing definition	Classification	Key considerations/challenges	Key findings/notes
Ahuja et al. [16]	2016	Not explicitly defined	Emphasis on "expeditious care"	Early decompression improves outcomes	Reinforces urgency in SCI management
Balain [17]	2017	Variable; context-dependent	Patient- and injury-specific timing	Surgery is influenced by physiological stability and injury characteristics	Highlights clinical variability
Thomas et al. [18]	2022	≤8 hours	Ultra-early	Investigated as a new standard	May offer superior clinical outcomes
Wilson & Fehlings [19]	2011	<24 hours	Early	Widely used threshold in research	Practical cutoff in many studies
Wilson et al. [20]	2020	8-12 hours (ultra-early), <24 hours (early), 48–72 hours (late)	Multi-tier classification	Benefits are greatest with early interventions	Time windows systematically analyzed
Furlan et al. [21]	2013	Not fixed; benchmarking study	Process-focused evaluation	Delays due to transfers, diagnostics, and patient variability	Identifies systemic and logistical barriers

Practical challenges complicate “early” surgery standardization despite these varied definitions. Differences in patient status, logistical constraints, and referral systems can result in delays that preclude meeting the ideal time frames described in controlled study environments. Wilson et al. systematically compared thresholds, including ultra-early (eight to 12 hours), early (24 hours), and late (48-72 hours) interventions, and observed that the benefits of decompression are maximized when surgery is performed at the lower end of these windows [20]. However, real-world factors may prevent such timely access to care. Furthermore, Furlan et al. offer a benchmarking appraisal of the surgical decompression process, identifying critical barriers such as inter-hospital transfers, insufficient preoperative diagnostics, and the inherent variability of clinical presentation, all of which contribute to the ongoing debate and challenge of standardizing early surgical intervention [21].

Influence of Surgical Timing on Neurological and Functional Outcomes

Based on data from multiple large clinical studies and meta-analyses, early surgical intervention in cases of acute SCI has been linked to notable improvement in neurological recovery. The "time is spine" concept emphasizes the benefits of ultra-early surgical decompression, often within 12 to 24 hours, in minimizing secondary injury processes, which can lead to improvements in motor scores and conversion of the ASIA grade [22]. Clinical guidelines stress that timely surgical stabilization is critical for preserving neurological function, as demonstrated by studies indicating that early decompression enhances the probability of ASIA grade conversion and yields higher motor recovery scores [23, 24]. If there was at least a one-grade improvement in AIS at the six-month follow-up, the neurological outcome of this study was considered "improved neurologic outcome." At the six-month follow-up, an ASIA grade of E was considered complete recovery. At the six-month follow-up, an ASIA grade of B, C, or D was considered incomplete recovery. If the ASIA grade did not improve from admission to the six-month follow-up, no recovery was considered to have taken place. The Barthel index score of 0-20 was used to evaluate the functional state or level of dependency at six months, with 0-60 representing total dependence, 21-60 representing severe dependence, 61-90 representing moderate dependence, and 91-99 representing mild dependence [24]. The idea that prompt surgical intervention slows down ongoing neurological degeneration and creates an environment that is favorable for brain regeneration or preservation is supported by thorough assessments of clinical evidence [25].

Aside from neurological recovery, early surgical intervention appears to favorably influence additional functional outcomes and healthcare resource utilization in patients with acute SCI. Timely surgery not only improves functional independence by accelerating rehabilitation outcomes, especially in polytrauma patients with multiple co-injuries, but also contributes to shorter intensive care unit and hospital stays, which can reduce the overall incidence of complications and mortality rates [23]. Early SCI management, which has been supported by robust clinical evidence and meta-analyses, is advantageous in two ways: it optimizes neurological recovery through earlier ASIA conversions and improved motor scores; and increases long-term rehabilitation and functional independence while reducing healthcare costs [24,18]. This supports the imperative inter-relationship between the timing of surgery, neurological recovery, and overall patient outcomes during the acute phase of SCI management (Table 3).

**Table 3:** Impact of surgical timing on neurological and functional outcomes in acute SCI SCI: Spinal cord injuries

Study	Year	Timing of surgery	Neurological outcome (e.g., ASIA conversion, motor score)	Functional outcome (e.g., independence, ICU/hospital stay)	Key findings/notes
Sandarage [22]	2025	≤12-24 hours	↑ ASIA conversion rate	Not specified	Emphasized the "time is spine” concept
Quinones et al. [23]	2024	Early vs. delayed	↑ Motor scores, ↑ ASIA conversion	↓ ICU/hospital stay	Early surgery improves both neuro and functional outcomes
Purkayastha et al. [24]	2023	≤24 hours vs. >24 hours	↑ Neurological recovery	↑ Functional independence, ↓ complications	Meta-analysis data supporting early decompression
Marland et al. [25]	2024	Not specified	↓ Neurological deterioration	Not specified	Supports rapid decompression to preserve neural tissue

Surgical Timing and Influence on Systemic Complications

Early surgical intervention in the management of traumatic SCI is associated with improved neurological outcomes and a potential reduction in systemic complications that extend hospital stays and increase healthcare resource utilization. Extended ICU stays and associated high costs have been linked to postoperative complications across various surgical fields [26]. In the specific context of SCI, Wilson et al. developed a clinical prediction model that categorizes complications into cardiopulmonary, thrombotic, infectious, and decubitus ulcer groups, showing that timely decompressive surgery may help mitigate the development of some of these adverse events [27]. Of the 376 patients enrolled in the study, the functional independence measure (FIM) motor score evaluations were available for 310 individuals at one year and for 66 patients at six months. Patients included and those omitted owing to insufficient follow-up did not differ significantly in initial ASIA impairment scale (AIS) grade, ASIA motor score (AMS), or MRI intramedullary signal characteristics (p > 0.05). Of the patients in the analysis, 358 (96.5%) had decompressive surgery, and 227 (62.1%) were given steroids. The percentage of steroids administered or the mean time to surgery did not differ significantly between patients who were included and those who were omitted because of insufficient follow-up (p>0.05). The mean FIM motor score at follow-up was 62.9 (-28.6), with successively lower entry AIS grades showing progressively higher mean scores [27]. Early surgical intervention may help shorten ICU stay and total hospital stay by lowering the occurrence of such problems, thereby optimizing the use of healthcare resources.

The rate of systemic complications after SCI surgery is largely influenced by multidisciplinary interventions in the perioperative setting. Early interdepartmental cooperation between the intensive care, surgical, and rehabilitation departments to avoid ICU-acquired complications such as pressure ulcers, ventilator-associated pneumonia (VAP), and deep vein thrombosis (DVT) is necessary [28]. Ventilator-associated pneumonia has been shown to prolong time-to-extubation and extend ICU stay significantly, which enhances total morbidity and cost [29]. Moreover, pressure ulcers are dangerous to patients' quality of life and recovery and represent serious long-term problems in neurosurgical settings [30]. Cumulatively considered, these results confirm the necessity for active multidisciplinary care programs and optimal surgical timing for diminishing both short- and long-term postoperative problems in SCI patients (Table 4).

**Table 4:** Surgical timing and systemic complications in acute SCI SCI: Spinal cord injuries

Study	Year	Type of complication	Influence of surgical timing	Role of multidisciplinary care	Key findings/notes
Alwaqfi et al. [26]	2025	General postoperative (ICU-related)	Early surgery reduces prolonged ICU stay	Not specified	High ICU costs linked to complications across surgeries
Wilson et al. [27]	2012	Cardiopulmonary, thrombotic, infectious, pressure ulcers	Early decompression linked to lower complication risk	Not specified	Developed prediction model for systemic complications
Wang et al. [28]	2022	DVT, VAP, pressure ulcers	Indirect reduction through early intervention	Emphasizes early team collaboration	Multidisciplinary care lowers ICU-acquired complications
Turkistani et al. [29]	2024	Ventilator-associated pneumonia (VAP)	Not directly addressed	ICU team involvement critical	VAP prolongs extubation, increases morbidity and cost
Vasselli et al. [30]	2022	Pressure ulcers	Not directly addressed	Critical role in long-term recovery	Pressure ulcers impair quality of life, prolong recovery

Expected Outcomes in Anterior Versus Posterior Surgical Spine Approach

Because the decision between the anterior and posterior approach can significantly impact clinical outcome, the surgical treatment of SCI continues to be a significant part of spine surgery (Table 5). Because it provides direct decompression of the spinal cord and neural elements, the anterior approach is typically chosen when the underlying pathology is one of direct compressive forces from anterior structures, i.e., osteophytes or herniated discs. To promote functional recovery, Zhao et al. mentioned that anterior decompression with internal fixation has the potential to directly remove ventral compressive tissues, restore intervertebral height, and reconstruct cervical lordosis [31]. In addition, Jiang et al. reported improved functional recovery and fewer complications in thoracolumbar fracture patients following anterior decompression surgeries, emphasizing the advantage of direct decompression in minimizing secondary neural damage [32].

**Table 5:** Comparison of anterior versus posterior approaches in the surgical management of SCI SCI: Spinal cord injuries, ACCF: Anterior cervical corpectomy and fusion

Study	Surgical approach	Indications/pathology	Advantages	Disadvantages/complications	Key findings
Zhao [31]	Anterior	Anterior compression (e.g., herniated discs, osteophytes)	Direct decompression, restoration of intervertebral height, and cervical lordosis	-	Improved functional recovery through direct ventral decompression and internal fixation
Jiang et al. [32]	Anterior	Thoracolumbar fractures	Superior functional outcomes, fewer complications	-	Anterior decompression is superior in minimizing secondary neural damage
Prasada & Ridia [33]	Posterior	Multilevel compression or intact anterior column	-	Increased blood loss, prolonged hospitalization	Posterior approach associated with more perioperative morbidity compared to anterior approach
Yao et al. [34]	Posterior	General SCI cases	-	Inadequate for anterior compression	Posterior decompression may be insufficient alone for anteriorly based pathology
Vasan [35]	Anterior/ combined	Cases with posterior ligamentous injury	Combined approach prevents kyphotic deformity	-	Anterior fusion alone risks kyphosis; combined approach may be necessary
Tatter et al. [36]	Anterior (ACCF)	Traumatic cervical injuries	Safe and effective if proper patient selection is made	-	ACCF outcomes depend heavily on surgical expertise and indication
Qian et al. [37]	Anterior	Cervical corpectomy cases	-	Anterior migration of spinal cord post-surgery	Highlights the risks and technical challenges of anterior decompression
Lee et al. [38]	Posterior (or combined)	Locked facets or posterior disruption	Ensures full decompression and stability	-	Anterior-only approach may be inadequate in cases with posterior pathology

However, the posterior approach has traditionally been used in situations where the compressive lesions are posterior, extend across multiple levels, or in situations where anterior column integrity is maintained. Systematic reviews such as the one by Prasada and Ridia highlight that the posterior approach tends to be associated with increased blood loss and longer hospital stays compared to the anterior approach [33]. Yao et al. corroborated such evidence by stating that posterior decompression alone will not be sufficient to alleviate the anterior compressing forces; therefore, it may produce suboptimal neurological outcomes in certain situations [34]. With a comparative observation between two distinct patient groups, the observation group had considerably longer operative times, intraoperative blood loss, and postoperative hospital stays compared to the control group (p<0.05). Despite the extra surgery burden, a better radiographic outcome was displayed in the observation group, with highly satisfactory restoration of the anterior and middle column vertebral height and additional correction of the Cobb's angle in the postoperative stage (P<0.05). Additionally, the observation group saw a considerably faster recovery of lower-extremity sensory and motor function during follow-up (p<0.05), indicating a more positive neurological recovery. In comparison to the control group, the observation group's total ASIA scores were significantly higher right after surgery, as well as six months and a year later, suggesting a more noticeable and long-lasting improvement in neurological function (p<0.05) [34].

The trade-offs between these two approaches have provoked investigations of combined surgical approaches when either method in isolation might not be sufficient to address all the biomechanical and decompressive requirements. Vasan et al. described how anterior fusion in isolation could place patients at risk for progressive kyphotic deformity, particularly in the setting of concomitant posterior ligamentous disruption, and thus promoted combined anterior and posterior stabilization techniques [35]. Tatter et al. also documented evidence that an anterior cervical corpectomy and fusion (ACCF) remains a safe and effective intervention for the treatment of SCI, further illustrating that outcomes of the anterior technique significantly depend upon proper patient selection and the surgeon's experience [36].

Unilateral complications unique to the anterior approach have also been described. Anterior spinal cord migration following ACCF has been described by Qian et al. as an event indicating technical severity and morbidity of ventral decompression [37]. Lee et al. explained that instances where locked facets or posterior element violation are present may require posterior treatment in an attempt to properly reestablish stability and decompression, and that an anterior-only approach may not be sufficient [38].

Combined, the evidence shows that while the anterior approach can achieve better direct decompression and allow for better early neurological recovery when anterior compressive pathology is the predominant feature, the posterior approach may be better in multilevel injury or injury with predominantly posterior elements. Lastly, the optimal surgical strategy for SCI includes meticulous regard to patient-specific morphology of injury, treatment timing, and skill of the surgeon, often yielding personalized treatments that combine both anterior and posterior methods to realize the best neurological recovery and spinal stability [31-38].

Practical and Ethical Considerations

The feasibility and timeliness of acute surgical therapy for SCI are heavily dependent upon resource limitations in low- and middle-income countries (LMICs). Inavailability of operating rooms as well as diagnostic equipment such as MRI can cause delays in starting potentially useful early decompression therapy [39]. The requirement of proper preoperative stabilization and inter-hospital transfer practices contributes to these structural barriers. In addition, implementation of evidence-based clinical practice guidelines in resource-limited environments is a challenge that requires tailor-made strategies taking into account local logistics and infrastructure capabilities [40]. In resource-limited environments, such adjustments must be made to guarantee that early surgery clinical benefits are realistic and feasible.

Decision-making during the acute phase of the care of SCI is made more difficult by ethical issues, particularly in dealing with resource scarcity. The principles of beneficence and non-maleficence must be weighed against the principles of justice and cost-effectiveness in deciding on acute surgery. Whether or not early surgery is a fair utilization of resources is a relevant ethical question in LMICs, where the healthcare system frequently functions on tight budgets [41]. Whether or not surgery is necessary in the case of life-altering injury should also be weighed regarding long-term factors, including its effect on rehabilitation and long-term comorbidity profiles, and any opportunity costs. An open framework needs to be created to coordinate ethical decision-making processes with the analysis of costs to obtain the most favorable results for patients and fairly distribute the use of the restricted surgical resources [40].

Future Directions and Research Gaps

Conducting well-designed randomized controlled trials (RCTs) that strongly investigate the effect of the timing of surgery on neurological and functional outcomes should be the top priority of future research in the surgical treatment of acute SCI. According to the Surgical Timing in Acute Spinal Cord Injury Study (STASCIS) trial, early surgery within 24 hours after injury might best optimize outcomes and demonstrate evidence for the advantages of early decompression surgery. But its findings also emphasize the importance of additional controlled trials that can eliminate residual heterogeneity and patient response uncertainties as defined by Fehlings et al. [3]. Along with improved trial design, further bibliometric analysis has demonstrated changing research topics of interest within the field, signifying the need for newer imaging modalities and biomarkers in study designs to reach a deeper understanding of injury mechanisms and the window period for intervention [42].

In addition to traditional clinical trial paradigms, there is a growing consensus on the importance of personalized approaches to optimize acute SCI care. The increasing sophistication of quantitative imaging techniques, as highlighted in translational reviews, provides a promising avenue for the development of reliable biomarkers that could serve as decision-making aids in determining surgical timing [43]. Additionally, triage systems based on artificial intelligence (AI) are becoming more and more potent instruments that can combine multi-modal patient data, such as neuroimaging and clinical profiles, to forecast results and customize surgical procedures for each patient. For such novel techniques to have therapeutic usefulness and predictive accuracy that are both reliable and generalizable, they must undergo thorough validation in prospective trials.

Multicenter partnerships and long-term registry data are also essential for the field's advancement as they offer the framework required to evaluate the sustainability of early surgical advantages over long follow-up times. The Rick Hansen SCI Registry is one example of a national and international registry that has already proven to be useful in gathering longitudinal outcome data that can guide clinical practice and policy [10]. To close current research gaps and open the door for evidence-based, individualized management strategies, these cooperative efforts are crucial for improving current treatment protocols as well as for discovering new outcomes and care pathways that could further improve the quality of life for people with SCI.

## Conclusions

The current body of literature supports early decompression surgery, ideally within 24 hours of injury, as a central pillar in the management of acute SCI. This timing is instrumental in enhancing neurological recovery and reducing complications, although successful outcomes also depend on the co-injuries of the patient and whether he/she will be fit to undergo surgery. Patients with a complete SCI, such as the ASIA A, are less likely to achieve any improvement in their neurological function.
